# Prevalence of bacterial vaginosis among pregnant women attending antenatal care in Tikur Anbessa University Hospital, Addis Ababa, Ethiopia

**DOI:** 10.1186/1756-0500-7-822

**Published:** 2014-11-20

**Authors:** Zemenu Mengistie, Yimtubezinash Woldeamanuel, Daniel Asrat, Addis Adera

**Affiliations:** Department of Medical Microbiology, Mizan Tepi University, P.O. Box 260, Mizan, Ethiopia; Department of Medical Microbiology, Immunology and Parasitology, Addis Ababa University, P.O. Box 9086, Addis Ababa, Ethiopia; Department of Nursing, Woldia University, P.o.Box 400, Woldia, North Wollo Ethiopia

**Keywords:** Bacterial vaginosis, Pregnancy, Prevalence

## Abstract

**Background:**

Bacterial vaginosis is one of the most common genital tract infections among reproductive age group. The prevalence of bacterial vaginosis varies from country to country even in the same country it varies among populations of interest. Different social and sexual factors can contribute to the development of bacterial vaginosis. The aim of this study was to determine the prevalence of bacterial vaginosis and to identify the possible risk factors associated among pregnant women attending antenatal care in Tikur Anbessa University Hospital, Addis Ababa, Ethiopia.

**Methods:**

Randomly selected 57 symptomatic and 195 asymptomatic pregnant women aged between 18 and 40 years visiting obstetric and gynecological clinic from November 2011 to April 2012 screenedusing Gram stain Nugent scoring system. Statistical analysis like univariate analysis to calculate frequencies and proportions, bivariate analysis to see association of selected exposure variables with the outcome variable, and multivariate analysis to check the association of possible factors with bacterial vaginosis by adjusting potential confounding factors was calculated using SPSS (Version 16.0).

**Results:**

The prevalence of bacterial vaginosis is 19.4% using Gram stain Nugent scoring system. In addition, prevalence of bacterial vaginosis is 31.6% and 15.9% among symptomatic and asymptomatic pregnant women respectively. A high percentage of bacterial vaginosis positive pregnant women were asymptomatic (63.3%). 36.7% bacterial vaginosis positive pregnant women reported abnormal vaginal discharge with or without unpleasant smell. Multiple lifetime sexual partner (OR: 8.6; 95% CI: 2.5, 29) and previous history of spontaneous abortion (OR: 5.9; 95% CI: 1.5, 23) had remained significantly associated with prevalence of bacterial vaginosis.

**Conclusion:**

The prevalence of bacterial vaginosis is higher among asymptomatic pregnant women and associated with the factors previous history of multiple lifetime sexual partner and spontaneous abortion.

## Background

Bacterial vaginosis (BV) is a shift in the vaginal ecosystem characterized by an overgrowth of anaerobes, and a decrease in Lactobacillus resulting in degradation of the natural flora that helps keep the vaginal tissue healthy [1]. It is a very common infection in women, and there is a lack of understanding regarding the triggers and factors for the onset and resolution of it [2].

Bacterial vaginosis is an important gynecologic problem of childbearing age group of women worldwide. The presence of bacterial vaginosis has consistently been shown to be a risk factor for adverse obstetric outcomes such as preterm labor and delivery, preterm premature rupture of membranes, spontaneous abortion, and postpartum infections such as endometritis and caesarean section wound infections [3–5]. It also increases the risk of HIV acquisition by approximately 60 percent; because BV increases HIV genital shedding with in discharge and results in increased concentration of HIV in genital secretions, which in turn facilitates both vertical and sexual HIV transmission [6–8].

The prevalence rates of bacterial vaginosis among pregnant women vary from 6.4 percent to 38 percent [9, 10]. It has been found that the lower the socioeconomic status of the population, the higher the incidences of bacterial vaginosis, which may indicate health and hygiene factors, play an even bigger role than anticipated [11]. The methods of diagnosis used for diagnosis ofBV can also have an effect on the variation of BV prevalence. The gold standard for diagnosis of BV is microscopic criteria proposed by Nugent [12]. The clinical criteria by Amsel do not require laboratory facilities, specialized staff and there is no delay in reporting. However, it is difficult to evaluate all of these criteria for diagnosis of BV in busy practice; and it requires the ability of the gynecologist to analyze wet mount microscopy.

Although many studies of BV have been done in different country,currently we know of no published studies that have been conducted in Ethiopia to describe the prevalence of BV among pregnant women. In order to avoid the above aforementioned pregnancy complications during BV infection screening and treating of pregnant women is crucial. Therefore, the present study was carried out to determine the prevalence ofbacterial vaginosis and associated factors among pregnant women visiting antenatal care (ANC).

## Methods

Fifty seven symptomatic and 195 asymptomatic pregnant women aging 18 to 40 years included in the study by simple randomsampling technique. These were outpatient clients visiting obstetrical and gynecological clinical for antenatal care services of a tertiary level hospital in Addis Ababa from November 2011 to April 2012. The nurse interviewers performed a comprehensive review of the prenatal and ultrasound record; and completed the standardized baseline questionnaire containing information regarding maternal age, gravidity, previous pregnancy outcomes, demographic, vaginal hygiene and contraceptive use. Information on the presence and type of vaginal discharge, history of sexual transmitted infection or genital tract infection, history of BV, vaginal douching practices and sexual practices also collected using questioner. The questionnaires filled in a confidential location within the office by a female nurse interviewer. Vaginal bleeding and antibiotic treatment in the preceding two weeks used as exclusion criteria.

After physical and obstetrical examination by the attending physician or obstetrician vaginal sample was collected from lateral wall of the vagina and then smeared on a slide and transported to microbiology department for Gram staining for Nugent scoring. This is a standardized 0–10 point scoring system for evaluation of Gram stained vaginal smears based on three morphotypes: large gram positive rods (lactobacilli), small gram negative/variable rods (G*.* vaginalisand anaerobic rods) and curved gram variable rod (Mobiluncusspecies). A score of 0–3 is considered normal, 4–6 intermediate, 7–10 positive for BV [12].

Data entered using EPI data then exported to SPSS version 16.0 for analysis. Statistical analysis like univariate analysis to calculate frequencies and proportions, bivariate analysis to see association of selected exposure variables with the outcome variable, and multivariate analysis to cheek the association of possible factors with bacterial vaginosis for adjusting potential confounding factors calculated using SPSS.

The Research and Ethical Review Committee (REC) of Addis Ababa University approved the research project proposal. Written informed consent obtained from willing pregnant women and symptomatic pregnant women having BV positive treated by 500 mg oral metronidazole twice daily for seven days.

## Results

The mean age of the study participant was 27.6 (±4.7)years and all are resident in urban. In addition nearly all 241(95.6%) of the study participants were married and 201(79.8%) had only one lifetime sexual partner. Among 161(63.9%) multigravida pregnant women, 24 (14.9%) of pregnant women had previous history of spontaneous abortion. None of the study participants had vaginal douching practice in the preceding month.

The Gram stain result of 183(72.6%) study participant classified as normal, 20 (7.9%) participant diagnosed as an intermitted while the remaining 49 (19.4%) were positive for bacterial vaginosis by Nugent scoring system. As shown in Table 1, the prevalence of bacterial vaginosis among symptomatic pregnant was 18(31.6%) but among asymptomatic the prevalence of BV was 31(15.9%). Majority (63.3%) of Bacterial vaginosis diagnosed pregnant women were asymptomatic.

**Table 1:** **Prevalence of bacterial vaginosis based on Nugent scoring system among pregnant women attending ANC in Tikur Anbessa university hospital (November 2011 - April 2012)**

	Microbiological diagnosis			Odd ratio	p- value
	***Positive no. (%)***	Negative no. (%)	Total no. (%)		
**Symptomatic**	18 (36.7)	39 (19.2)	57 (22.6)	2.44	< 0.05 (0.032)
**Asymptomatic**	31 (63.3)	164 (80.8)	195 (77.4)	1.00	
**Total**	**49 (19.4)**	**203 (80.6)**	252 (100**)**		

More vaginal symptoms were reported from BV negative pregnant women than BV positive pregnant women but abnormal discharge with unpleasant smell was reported more frequently by BV positive women. Out of unusual vaginal discharge complained women only 27 (47.4%) had non-Candida like discharge and non-Candida like discharge is significantly associated with BV.

In the Bivariate analysis (Table 2) personal monthly income ≤500 ETB, multiple sexual partners history, first trimester gestational age and previous history of spontaneous abortion, daily showering were associated with bacterial vaginosis(P < 0.05). However, after we had adjusted for confounders in multivariate analyses (Table 2), more than one lifetime sexual partners and previous history of spontaneous abortion remained independently associated with increased likelihood of BV positive. Some factors that lacked independent associations with BV during pregnancy remained important confounders and were therefore retained in the final model.

**Table 2:** **Univariate & Multivariate analysis of factors association with bacterial vaginosis among pregnant women attending ANC in Tikur Anbessa Hospital (November 2011 - April 2012)**

Variables	BV positive	BV negative	Univariate; COR (95% CI)	Multivariate; AOR (95% CI)	p-value
**Age (year)**					
≤20	3(17.6)	14(82.4)	1.09(0.27, 4.33)		
21-29	33(21.2)	123(78.8)	1.36(0.67, 2.77)		
30+	13(16.5)	66(83.5)	1.00		
**Religion**					
Orthodox	40(22.2)	140(77.8)	1.00		
Muslim	7(13.7)	44(86.3)	0.56(0.23, 1.33)		
Protestant	2(9.5)	19(90.5)	0.37(0,08, 1.65)		
**Education**					
< Grade 12 complete	25(23.1)	83(76.9)	1.51(0.81, 2.82)		
≥ Grade 12 complete	24(16.7)	120(83.3)	1.00		
**Occupation**					
Employed	19(18.1)	86(81.9)	1.00		
House wife	24(20.3)	94(79.7)	1.16(0.59, 2.26)		
Others	6(20.7)	23(79.3)	1.18(0.42, 3.3)		
**Income (ETB)**					
≤500	29(31.5)	63(68.5)	*5.26(2.16, 12.8)	3(0.6, 14.6)	0.17
501-1500	13(17.8)	60(82.2)	2.48(0.93, 6.58)		
>1500	7(8.0)	80(92.0)	1.00		
**Number of LTSP**					
One	20(10)	181(90)	1.00		
Two and above	29(56.9)	22(43.1)	*11.9(5.8, 24.5)	8.6(2.5, 29)	0.01*
**Gestational age**					
1st trimester	13(36.1)	23(63.9)	*3.46(1.48, 8.1)	1.9(0.4, 8.6)	0.40
2nd trimester	19(20)	76(80)	1.53(0.75, 3.14)		
3rd trimester	17(14)	104(86)	1.00		
**Number of pregnancy**					
Primigravidia	17(18.7)	74(81.3)	1.00		
Multigravidia	32(19.9)	129(80.1)	1.08(0.56, 2.08)		
**History of abortion**					
Yes-spontaneous	12(50)	12(50)	*6.28(2.45, 16.11)	5.9(1.3, 23)	0.012*
Yes-induce	2(33.3)	4(66.7)	3.14(0.54, 18.41)		
No	18(14)	113(86.3)	1.00		
**Vaginal bathing**					
Daily	46(18.8)	199(81.2)	0.31(0.07, 1.43)		
Less than daily	3(42.9)	4(57.1)	1.00		
**Showering frequency**					
Daily	16(57.1)	12(42.9)	*7.72(3.35, 17.78)	2.7(0.6, 12)	0.19
Less than daily	33(14.7)	191(85.3)	1.00		
**Previous BV/GTI**					
Yes	3(15)	17(85)	1.4(0.39, 4.99)		
No	46(19.8)	186(80.2)	1.00		
**Yeast infection**					
Yes	9(32.1)	19(67.9)	2.18(0.92, 5.17)		
No	40(17.9)	184(82.1)	1.00		

COR – Crude Odd Ratio; AOR – Adjusted Odd Ratio LTSP – Life Time Sexual Partner; ETB- Ethiopian Birr GTI- Genital Tract Infection; *statistically significant.

## Discussion

The overall prevalence of bacterial vaginosis by Gram stains Nugent scoring criteria in this study was 19.4%, which was comparable with studies done in India (20.5%) and in Denmark (17%) [13, 14]. The current study showed lower prevalence of bacterial vaginosis than reports from different sub-Saharan countries like Kenya (37%), Botswana (38%), Zimbabwe (32.5%) using the same methods of diagnosis [10, 15, 16]. The lower prevalence of BV in our study may be due to that absence of vaginal douching among study population. Besides these, the lower prevalence of BV in this study can be also explained as most of the women were in third trimester gestational age since as gestational age increase the prevalence of BV decrease. In contrast to the present study, lower prevalence of bacterial vaginosis was reported in Burkina Faso (6.4%), India (8.6%), Sweden (9.3%), Boston (11%) and Washington (12%) [9, 17–20]. This may be due to environmental, behavioral, socioeconomic status and stressor differences in the geographical variation.

Bacterial vaginosis is mostly present without sign and symptoms. The most common clinical sign and symptoms of BV is thin white or gray homogenous vaginal discharge with or without unpleasant smell. The smell of the discharge mostly noticed after sexual intercourse [3, 21, 22]. In the current study, we found that 63.3% of participants who were diagnosed positive for BV by gram stain had no symptom for BV. This result is consistent with other studies done in different countries [10, 23, 24]. In this study the result also showed that the Vaginal discharge complains by women has less value as diagnostic algorithm because approximately one-third (31.6%) of BV positive participants were only reported abnormal discharge when asked. The finding are consistent with other study which report that only very few women reported vaginal symptom when asked [15]. Comparing with a women having Candida like discharge, pregnant women with non-Candida like abnormal discharge had a greater chance (OR: 4.6; 95% CI: 1.4, 15.7; p = 0.014) to be positive for bacterial vaginosis.

In addition, our result also found that the presence of abnormal vaginal discharge (p = 0.01) and unpleasant smell (p = 0.005) were reported vaginal symptoms associated with bacterial vaginosis. In general abnormal vaginal discharge is not accurate sensitive indicator of BV status in this study which confirms the suggestions by other studies [10, 25]; but symptomatic pregnant women have more than a twofold increased chance to be positive for bacterial vaginosis than asymptomatic.

With consideration to the above findings, it can be concluded that bacterial vaginosis has a varying degree of prevalence rate among people of different communities which might be due to certain factors such as hygiene behaviors and sociodemographic characteristics. Therefore, it is important to try to establish a correlation between BV and factors affecting its prevalence. Because our sample population was exclusively urban living and nearly all women were married, had no previous history of preterm and stillbirth and had daily vaginal bathing, we could not effectively examine the association of BV with these risk factors. As well, we were unable to compare the frequency of bacterial vaginosis among religious denominations due to lack of comparability in the number of participants. In our study, there was a significant correlation between number of lifetime sexual partner and prevalence of BV. Multiple lifetime sexual partners were more likely risk factors for bacterial vaginosis. This is consistent with other researcher findings [3, 26–28]; while others finding showed no significant correlation in this regard [29]. In addition to this factor, consistent with other study done among pregnant women in Burkina Faso [9]; we found that previous spontaneous abortion is independently associated as a risk factor of bacterial vaginosis infection.

## Conclusion

The overall prevalence of bacterial vaginosis among pregnant women is 19.4%. The prevalence of BV among symptomatic pregnant women was 31.6% while among asymptomatic pregnant women, it was 15.9% and BV infection is mostly asymptomatic. Since common asymptomatic nature of BV infection, screening of women having an experience of multiple lifetime sexual partner and previous history of spontaneous abortion is vital for good pregnancy outcome.
